# Smartphone-based serious games for mental health: a scoping review

**DOI:** 10.1007/s11042-024-18971-w

**Published:** 2024-04-06

**Authors:** Águeda Gómez-Cambronero, Anna-Lisa Mann, Adriana Mira, Gavin Doherty, Sven Casteleyn

**Affiliations:** 1 GEOTEC Research Group, Institute of New Imaging Technologies, Universidad Jaime I, Castellon, Spain; 2 School of Computer Science and Statistics, Trinity College Dublin, Dublin 2, Dublin, Ireland; 3 Personality, Evaluation and Psychological Treatments Department, University of Valencia, Av. de Blasco Ibáñez, 13, 46010 Valencia, Spain

**Keywords:** Smartphone, Serious games, Scoping review, Mental health, Mobile health, Digital therapy

## Abstract

The use of smartphone-based Serious Games in mental health care is an emerging and promising research field. Combining the intrinsic characteristics of games (e.g., interactiveness, immersiveness, playfulness, user-tailoring and engaging nature) with the capabilities of smartphones (e.g., versatility, ubiquitous connectivity, built-in sensors and anywhere–anytime nature) yields great potential to deliver innovative psychological treatments, which are engaging, effective, fun and always available. This article presents a scoping review, based on the PRISMA (scoping review extension) guidelines, of the field of smartphone-based serious games for mental health care. The review combines an analysis of the technical characteristics, including game design, smartphone and game-specific features, with psychological dimensions, including type and purpose of use, underlying psychological frameworks and strategies. It also explores the integration of psychological features into Serious Games and summarizes the findings of evaluations performed. A systematic search identified 40 smartphone-based Serious Games for mental health care. The majority consist of standalone and self-administrable interventions, applying a myriad of psychological strategies to address a wide range of psychological symptoms and disorders. The findings explore the potential of Serious Games as treatments and for enhancing patient engagement; we conclude by proposing several avenues for future research in order to identify best practices and success factors.

**Supplementary Information:**

The online version contains supplementary material available at 10.1007/s11042-024-18971-w.

## Introduction

Globally, 970 million people (about 1 out of 8) are affected by mental health disorders, which are the leading cause of years lived with disability (YLDs), accounting for 15.6\% of all YLDs worldwide [1]. They cause significant distress and negatively impact different aspects of life, such as social relationships, education, physical health, employment, and overall, quality of life [2]. Psychological therapies have been demonstrated to be beneficial for people suffering from mental health disorders [3], but less than 25\% of those affected in lower-middle- and upper-middle-income countries and only about one-third in high-income countries receive treatment [4].

Several barriers to access were identified that explain this treatment gap. Mental health care is an underfunded segment of healthcare in many countries, with widespread difficulties relating to insufficient infrastructure, trained personnel, and specialized professionals with respect to demand, leading to long waiting lists and/or non-treatment [5–7]. Other barriers include the social stigma of mental disorders [8], as well as other attitudinal (e.g., low perceived need, cultural beliefs, internalized and self-stigma, shame) and structural (e.g., geographical challenges, cost, time-related restrictions, lack of insurance coverage) barriers [9–12].

In an attempt to reduce the aforementioned barriers, the use of Information and Communication Technology (ICT) to deliver psychological interventions has been actively researched. Internet-based psychological treatments, a form of distance psychotherapy delivered via the internet on interactive multimedia devices such as computers, tablets or smartphones, are seen as having the potential to improve temporal and geographical access to mental health services, lower attitudinal barriers (e.g., stigma, shame) [13] and are generally cost-effective [14], thereby reaching a wider public with lower costs and shorter waiting times [15].

Furthermore, Internet-based programs have been proven to be effective for the treatment of several mental health disorders, such as anxiety or depression [16–18], with some studies demonstrating comparable efficacy to traditional face-to-face therapy [16, 19].

Due to their new capabilities and increasing prevalence, internet-connected mobile devices – mainly smartphones – have fueled a new stream of technology-based interventions bundled under the Mobile Health (mHealth) denominator. Researchers are exploring novel approaches to the delivery of treatment which exploit the versatility, ubiquitous connectivity, built-in sensors and anywhere–anytime nature of smartphones [20].

Potential advantages of mHealth include continuous availability, accessibility beyond geographical boundaries, equity in access to mental health resources, immediate support, anonymity, personalized content, lower cost, and increased capacity and service efficacy [21]. Smartphones, for example, allow systematic and automated patient data collection, the use of additional features such as reminders, access to contextualized psychoeducational content, personalized feedback and momentary ecological interventions, whereby patients have access to customized psychological care, when and where they need it most [22, 23]. mHealth apps can serve as stand-alone self-management, guided or adjunctive treatments [24], whereby parts of the therapy are moved out of in-person sessions and into real-life situations [25]. Although preliminary evidence of efficacy for specific mental disorders is emerging [20, 24, 26, 27], studies in mHealth therapies are typically affected by high attrition rates and low adherence, which may threaten the validity of findings [28].

Gamification and by extension Serious Games, are promising solutions to increase user engagement, and therefore improve adherence to treatment and reduce attrition rates [29, 30]. Gamification is the application of gaming elements and techniques (e.g., scoreboards, competition, awards) in regular applications, while Serious Games are video games that not only have an entertaining purpose but also the objective to improve specific aspects of learning or training [31]. Characteristics such as immediate feedback, a playful nature, the capacity for mental distraction, engagement and challenge align well with psychological treatments [32]. In addition, Serious Games have the advantage of providing customization and flexibility to accommodate different scenarios, as players tend to adopt diverse gameplay strategies and styles based on their abilities and behavior [33].

Existing reviews [30, 32, 34–42] reveal a clear interest in the use of video games for mental health purposes. On one hand, there is a focus on uncovering the potential therapeutic benefits of using commercial games which were primarily intended for entertainment [34, 37, 38]. On the other hand, some (older) studies examine purposely developed Serious Games and gamification techniques in the context of mental health care [32, 35, 36, 43], yet they predominantly concentrate on desktop games, leaving the role of mobile video games under-explored, despite the increasing popularity of smartphone-based mental health interventions [20]. A more recent systematic review on Serious Games separates games by platform, including smartphone-specific games, yet focuses specifically on games for depression and anxiety [44]. Given the recently increased interest in research in mobile Serious Games for mental health [45–52] there is a need to analyze, structure and summarize the body of research in this emerging field.

Therefore, we conducted a scoping review to methodically map and report the status quo of the usage of smartphone-based Serious Games in mental health and to identify possible gaps in the literature. To reach this objective, the following research questions have been formulated:

1. Which types of games are being employed?
2. What are the psychological purposes (e.g., prevention, assessment, treatment, follow-up, or relapse-prevention) the game addresses and how is it used?
3. Which mental health disorders and symptoms do these interventions focus on?
4. Which psychological frameworks and which of their components are incorporated into the Serious Games, and in which way?
5. Which design process was used and who was involved?
6. Which of the smartphone's capabilities are used in the game?
7. What form of evaluation is performed and what is evaluated?

These research questions aim to cover both the psychological and technical dimensions of the research space and are in line with existing systematic reviews with similar purpose [20, 32, 34].

## Methods

This scoping review investigated the existing literature on the use of smartphone-based Serious Games in mental health care. The authors followed the guidelines of Preferred Reporting Items for Systematic Review and Meta-Analysis- Extension for Scoping Reviews (PRISMA-ScR) [53] to conduct this review. The protocol followed for this review was registered with the Open Source Foundation (https://osf.io/gxbzw).

### Search strategy

In order to cover computing, clinical and psychological research, the ACM Guide to Computing Literature, PubMed, and PsycInfo databases were used to retrieve articles. To identify further relevant articles, forward reference list checking was carried out for the included articles, as well as for related reviews on the use of video games for mental health care.

The search was conducted between December 1st, 2021, and December 6th, 2021, and was repeated on January 10th, 2023 to include the results of 2022. In addition, in January 2024, the searches were reiterated, refining the search query by incorporating previously missing, high-frequency keywords in the articles previously included.

### Search terms

For this review, a combination of mental health- and technology-related search terms was used. The search terms combined three main areas: technology (e.g., “Smartphone”), software (e.g., “Serious Games”), and psychological target (e.g., “Mental Health”). Queries were designed with keywords informed by previous reviews about video games and smartphone applications for mental health [20, 35, 36] and Medical Subject Headings (MeSH) Terms were incorporated in order to facilitate the search and extraction of the medical information from PubMed. A list containing the search queries used to search each database is available in Appendix Section 6.1.

### Study eligibility criteria

In order to be included in this review, studies had to focus on Serious Games designed to be used in mental health care. This includes the assessment, prevention, intervention, follow-up, or relapse-prevention of a specific mental disorder or symptom, or the improvement of mental well-being. Games originally designed for entertainment purposes are excluded. Further, the review focused on applications explicitly developed for or tested on smartphones. Research addressing any other form of intervention delivery was excluded. We restricted our search to articles published in international journals and to full research articles in conference proceedings. Articles that were not first-hand original research contributions, such as reviews or mapping studies, were excluded. Lastly, only articles written in English and which were accessible were included. No restrictions regarding the study design, measured outcome, country or year of publication were imposed.

### Study selection

The study selection process was carried out in two phases. In the first phase, articles were assessed based on their titles and abstracts. In order to standardize the interpretation of inclusion criteria, a subset (15\%) of the articles was examined independently by two authors. Disagreements were rectified through discussion and consensus. If no consensus was reached, a third reviewer was consulted. Afterwards, the remaining articles were split between the same two authors and screened. In the second phase, the full text of the articles which passed the first phase was reviewed. The same authors read and tested the articles against the eligibility criteria independently, and reasons for exclusion were recorded. Possible disagreements were again solved by discussion and consensus with the input of a third reviewer.

### Data extraction and data analysis

For all included studies, fifteen study characteristics were extracted along three axes: general aspects of the study (name of the Serious Game, brief description of the game concept, genre, evaluation type, and target), technological aspects (game design process, engagement techniques, smartphone’s capabilities used, and integration of psychological care elements), and psychological aspects (psychological purpose -i.e., assessment, treatment, prevention-, psychological target, psychological framework used, psychological strategies used, and type of usage in psychological care). The data extraction was carried out by the first author and verified by the other authors. A summary of the information related to the target characteristics was recorded and synthesized using a descriptive approach in a joint Excel document. The studies were grouped and classified based on their similarities into categories. A full version of the data extraction table is found as Supplementary Material.

## Results

**Fig. 1:**
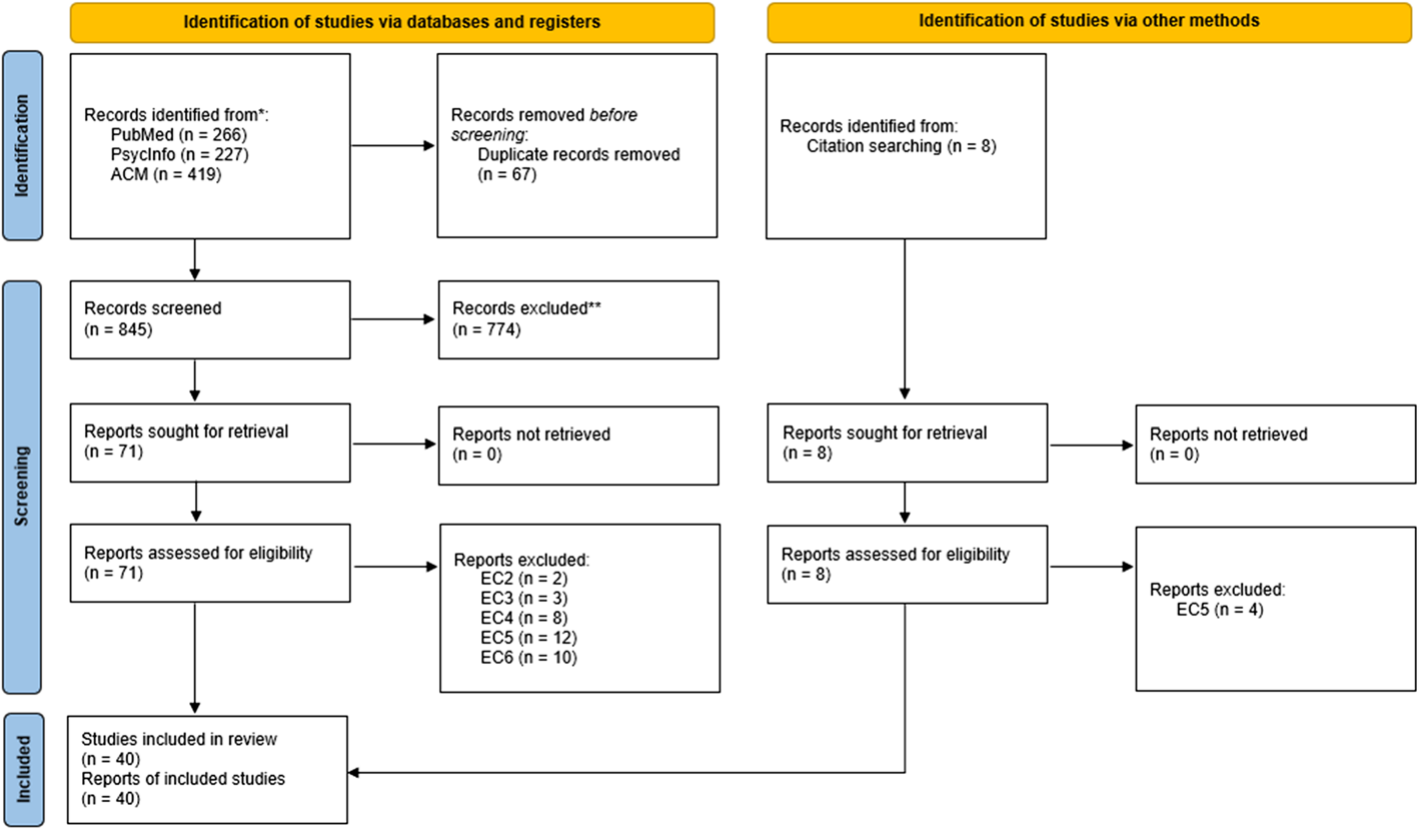
PRISMA 2020 flow diagram for systematic reviews which included searches of databases, registers and other resources

Figure 1 illustrates the search process undergone for this scoping review. Initially, 912 records were retrieved from the three selected scientific databases (see Section 2.1). After screening based on title and abstract, 71 were conserved. In this initial set of 71 retained articles, a citation search was performed, whereby all titles and abstracts of cited articles were screened, resulting in an additional 8 articles included. After reading the full article, 40 articles were included. Upon reiterating the searches with the updated query, a total of 552 new entries were scrutinised. Among these, 7 were retained following an assessment of their titles and abstracts, and subsequently, 3 were included after a comprehensive reading of the full articles, ensuring compliance with the inclusion criteria. Finally, 43 articles describe a total of 40 different Serious Games were included in this review (see Appendix Section 6.2 for a list of all games, identified by their name, and citation(s) to the corresponding article(s); 4 games without a name are identified by NM - Not Mentioned) + their citation-). All articles were published between 2014 and 2022. Out of the 43 articles included in this study, approximately 30\% (12 papers) were published in the last year (2022). Before that, the field was emerging, with small numbers of publications (yearly average of 4.77).

### Description and genre of the Serious Games

In this study, Serious Games have been classified using the major game genres specified by Adams (2014, p.67) [54]. Where the game genre is not explicitly stated, the classification has been based on the authors' descriptions and/or screenshots found in the corresponding articles. Upon reviewing the articles on each Serious game, it was found that the majority belong to the action and arcade game genre (14). The second most common genre is puzzle games (8). Other categories were Adventure (7), role-playing (4), simulation (4) and strategy (2). Appendix Section 6.3 overviews the 40 Serious Games, specifying their name, genre, and a short description of the game concept.

### Game design process

Information regarding the design process was attainable for a total of 22 Serious Games. For the remaining 18, neither the design process nor the individuals involved were reported. Among the design approaches that were identified, there were five designed based on the components of existing psychological protocols or theories (No Fume, RegnaTales, SPARX Japanese-adapted, SupperBetter, REACH). Thirteen Serious Games made use of participatory design (Grow It App!, Haddy, HAPPIA, HospiAvontuur, Legend of Evelys, LINA, Lumi Nova, MindMax, NM Samonte, et al. 2022, POD Adventures, The Guardians, Triumf, REACH), six games used iterative design (Gamified SmartCAT, LINA, Match Emoju, MindMax, POD Adventures, REACH), seven games employed user-centered design (Gamified SmartCAT, Grow It! App, Haddy, Lumi Nova, POD Adventures, Triumf, REACH), one game implemented a design based on theory (Raw Hand), and one reported only the use of prototypes (Magis). It should be noted that nine Serious Games made use of more than one of these design approaches (Gamified SmartCAT, Grow It App!, Haddy, LINA, Lumi Nova, MindMax, POD Adventures, Triumf, REACH).

Information regarding the people involved during the design process was provided for 17 Serious Games. Fifteen reported collaborating in multidisciplinary research teams. Health professionals (i.e. mental -psychologists and psychiatrists- and general health professionals) have been part of 14 out 15 of the multidisciplinary teams (Health e-Minds, Gamified SmartCAT, Grow It App!, Haddy, HAPPIA, HospiAvontuur, LINA, Lumi Nova, Match Emoji, NM Samonte, et al. 2022, POD Adventures, Raw Hand, Triumf, REACH). Moreover, the remaining two Serious Games which were not reported to be created by interdisciplinary teams were designed by health professionals (Legend of Evelys, Magis). In twelve of the Serious Games, the targeted end-users have been involved (Gamified SmartCAT, Grow It App!, Haddy, HospiAvontuur, LINA, Lumi Nova, Match Emoji, MindMax, NM Samonte et al. 2022, POD Adventures, Raw Hand, Triumf). The teams of ten Serious Games comprise various technical profiles such as software developers, analysts, game designers, or game industry experts. Furthermore, certain teams (HospiAvontuur, LINA, Lumi Nova, Math Emoji, MindMax, POD Adventures, REACH) comprise members with any of the following backgrounds: research, education, art, and playwright. Appendix Section 6.4 provides a detailed overview of the combinations of design approaches and people involved for each Serious Game.

### Smartphone capabilities used

Multimedia content such as images, sounds, audio, video, or animations is the most common capability used in all games. However, eleven games go further, using sensors such as accelerometers (NM Francillete et al. 2018, PuzzleWalk), Virtual Reality headsets (Serenity y NM Vara et al. 2016), cameras (Happify Breather, LINA, Grow It! App, Gamified SmartCAT), GPS (Gamified SmartCAT), microphone (SmokeFreeBrain), and biofeedback through other hardware (Relax and Race, The Loom, Haddy). Accelerometers are used to detect the user's physical activity. In particular, the game presented in Francillete et al. 2018, collected sensor data through the Google Activity Recognition API and converts it into a resource of the game. In the case of PuzzleWalk, the ActiGraph wearable accelerometer is used to detect daily walking steps, physical activity intensity, and sedentary time. Camera(s) are used to take photos (Grow It App!), for augmented reality (LINA), to receive biofeedback such as heart rate variability (Happify Breather), and to detect the context (Gamified SmartCAT). Biofeedback is also collected by smartwatches linked to the smartphone via Bluetooth (Haddy), and by a skin conductance biofeedback device (Relax and Race, The Loom). Microphone is used as game control to detect user's blow (SmokeFreeBrain). GPS is used in one Serious Game-supported treatment in order to detect if the user enters a specific area (Gamified SmartCAT). This technology is not directly used in the Serious Game, but is introduced in an exposure module within the encompassing psychological program.

### Purpose of psychological care

Out of the 40 Serious Games, 31 prioritize psychological intervention or treatment as their main focus. Nine Serious Games focus on preventing the development of a mental health problem, whether or not derived from a health condition. Two of the Serious Games can be used as both prevention and early intervention (POD Adventures, REACH). The remaining two Serious Games focus on psychological assessment (MyCQ, ACE). See Appendix Section 6.5 for more detail.

### Type of usage in psychological care

The included articles indicate that Serious Games serve two roles within psychological treatments. Certain games (or a set of mini-games) can function as standalone treatments or evaluations, while others serve as components within larger psychological treatment (i.e., these games do not constitute a complete treatment on their own). In addition, Serious Games-supported treatments can be self-administered or used as supportive tools for traditional therapy approaches. Of the 37 Serious Games reviewed, 34 are utilized as a standalone treatment or psychological evaluation, while the remaining 6 were used to target specific psychological strategies or modules of a more extensive therapy (e.g., reward/distraction –Serenity– or reframing negative thoughts –REACH–). All bar four (AquaSnap, MyCQ, Gamified SmartCAT, HAPPIA) can be self-administered. Two others indicate they can be used to complement conventional therapy (Raw Hand and RegnaTales).

### Psychological target

**Fig. 2:**
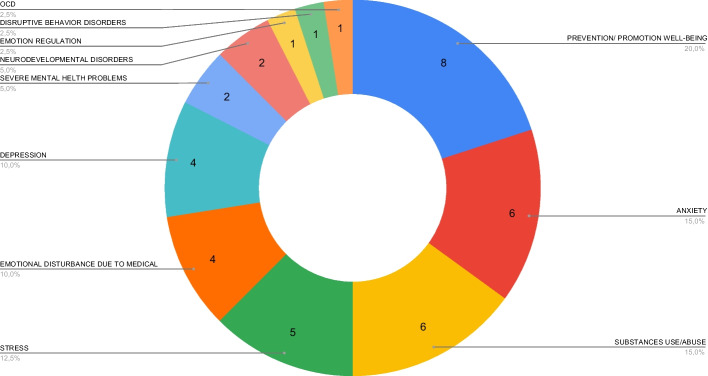
Psychological target addressed by Serious Games

The psychological target of these interventions vary widely as Fig. 2 displays. Prevention and promotion of well-being is the most prevalent objective among the corpus. Anxiety, substance use/abuse, and stress are the second most prevalent target. Within the substance use/abuse category, there are Serious Games that specifically focus on alcohol use (Drug Defense and JIB), and tobacco consumption (No Fume, SmokeFreeBrain), while the rest target the use of other substances (MyCQ and AquaSnap).

Other frequent targets include depression and emotional disturbance due to medical procedures or illness (i.e., Serious Games focusing on problems derived from COVID-19 –Magis, Haddy, and Legend of Evelys–, problems derived from surgical procedures –HospiAvontuur and Triumf–, and problems derived from an illness as breast cancer –I Love Breast–). Severe mental health problems, Neurodevelopmental Disorders, emotion regulation, Disruptive Behaviour Disorders, and Obsessive Compulsive Disorder (OCD) are less commonly targeted.

### Psychological framework used

The majority of articles, specifically 24, lack an indication of the applied psychological framework. Among those that report it, Cognitive Behavior Therapy (CBT) is the most used psychological framework, applied in studies related to 12 Serious Games. Three Serious Games are based on other psychological frameworks, namely Behavioral Activation, Positive Psychotherapy (PP) and Third Wave Therapy (3rd Wave) – including Acceptance and Commitment Therapy (ACT). Note that in some cases, different frameworks are combined (e.g., both PP and ACT are applied in MindMax).

For the remaining 24 Serious Games which do not specify the psychological framework used, some of them do mention specific theories, for example, Self-Determination Theory (Triumf) or Stress-Coping Theory (POD Adventures).

### Psychological strategies used

**Fig. 3:**
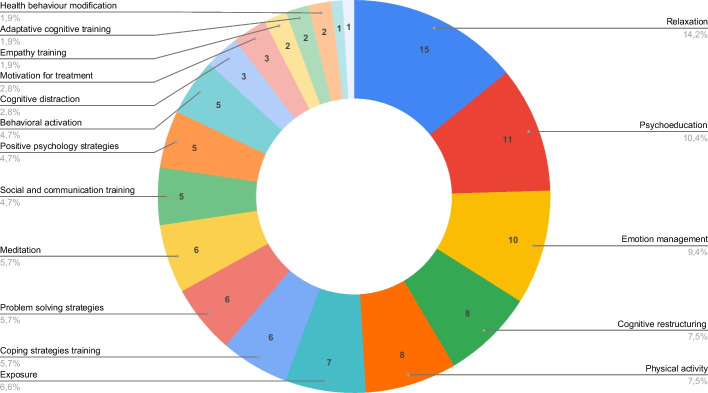
Distribution by psychological strategies used in the psychological programs found

The identified Serious Games deploy different psychological strategies to provide or support psychological care. Where the game was used as a standalone psychological treatment (34 out of 40—see Section 3.5), we consider the strategies from the Serious Game. For the remainder, as the authors made no distinction between techniques used in the game and in the full treatment, we consider all strategies used. As visible in Fig. 3, Relaxation, Psychoeducation, and Emotion management account for approximately one third of the psychological strategies used. The techniques associated with each Serious Game as well as the corresponding articles are listed in Appendix Section 6.6. It should be noted that 17 Serious Games utilized more than one psychological strategy, while the remaining games incorporate only a single psychological strategy.

### Integration of psychological strategies

In order to classify how the psychological strategies are incorporated into the Serious Games, ten categories have been established based on the game elements outlined by Adams [54]. The following classification was used:

- **Challenge:** psychological strategies are mandatory actions, which require an active effort of the player, to progress in the game.
- **Action:** applying the psychological strategies is an available action in the game, without being mandatory to progress.
- **Gameplay:** the psychological strategies are incorporated as both challenges and actions, since gameplay consists of facing challenges through actions to achieve the objective of the game.
- **Feedback:** the psychological strategies are integrated as a result of a player's actions either by modifying elements that affect the gameplay (such as speed or time) or by directly appearing in the game (e.g., text, video, animations).
- **Economy:** the psychological strategies are part of the internal economic system (i.e., currency or resource received in order to be able to carry out another action and/or progress in the game).
- **Quest:** the gameplay is divided into missions, whereby each has the objective to put a psychological strategy into practice.
- **Aesthetics:** any visual and auditory aspect in the game is closely related to a psychological technique.
- **Story:** the narrative or evolution within the game explains, tells, or is related to the psychological content.
- **Extra information:** psychological content -independent of the game story- is presented in between levels or scenes.

Table 1 displays which psychological techniques are integrated into which Serious Game.

**Table 1:** Integration of psychological strategies

Game and articles	CH	AC	GA	FE	EC	QU	AE	ST	EI
ACE			X						
AquaSnap			X						
Drug Defense			X					X	
EVO Project			X						
Gamified SmartCAT			X				X		
Grow It! App				X			X		
Haddy	X						X		
Happify Breather			X				X		
Healthy-e Minds				X					
HospiAvontuur			X				X	X	
I Love Breast			X				X	X	
JIB			X	X			X	X	
Journey to the west			X				X	X	
Legend of Evelys			X				X	X	
LINA								X	
Lumi Nova			X			X			
Magis								X	
Match emoji							X		X
MindMax		X		X					
MyCQ			X						
NM Vara, et al. (2016)			X						
NM Francillette, et al. (2018)		X			X				
NM Fraiwan, et al. (2015)			X		X		X	X	
NM Samonte, et al. (2022)			X				X		
No Fume			X	X			X	X	
Personal Zen			X				X		
POD Adventures			X			X	X		X
PuzzleWalk	X								
Raw Hand			X				X		
REACH			X				X		
RegnaTales			X			X			
Relax and Race			X				X		
Serenity		X							
SmokeFreeBrain			X				X		
SPARX (Japanese-adapted)			X			X	X	X	
SupperBetter		X				X	X		
The Guardians	X				X	X			
The Loom			X				X		
Triumf			X	X			X	X	

*CH* Challenge, *AC* Action, *GA* Gameplay, *FE* Feedback, *EC* Economy, *QU* Quest, *AE* Aesthetics, *ST* Story, *EI* Extra-information

### Engagement techniques

The engagement techniques have been extracted based on intrinsic motivation theories [55, 56] and the role of engagement in playing video games [57, 58]. The following classification was used:

- **Challenge:** well-defined goals which are not guaranteed to be accomplished, due to the utilization of techniques such as randomness, hidden information, variable difficulty levels, or multi-level goals [55].
- **Curiosity:** attraction to changes in the environment (sensory curiosity) or desire to do better (cognitive curiosity) [55].
- **Fantasy:** creation of learning experiences in an imaginary and fictional world in which curiosity and imagination are promoted, encouraging the users in the learning process [55].
- **Autonomy:** ability to make decisions [56].
- **Competence:** ability to apply skills and acquire mastery [56].
- **Relatedness:** creation of social connections [56].
- **Feedback:** introduction of immediate feedback to create a responsive and therefore interestingly complex environment [55].

Additionally, engagement factors in online treatments [59–62] and extrinsic motivation have been taken into account in the categorization.

- **Rewards:** promoting extrinsic motivation through points or prizes.
- **Self-assessment:** ability to register and monitor achievements (e.g., drug intake, physical exercise) and symptoms, with access to comprehensive reporting and progress updates.
- **Support:** any type of assistance or supplementary support provided during the Serious Game-supported treatment.

Table 2 shows the mapping between engagement techniques and Serious Games, ordered from the most to the least incorporated engagement technique.

**Table 2:** Engagement techniques incorporated in the Serious Games

Engagement technique	Serious Game
Rewards (17)	REACH, Personal Zen, Relax and Race, SupperBetter, I Love Breast, Gamified SmartCAT, Triumf, NM Fraiwan et al. (2015), MindMax, NM Francillette et al. (2018), Healthy-e Minds, Haddy, Grow It! App, Lumi Nova, PuzzleWalk, AquaSnap, The Guardians
Autonomy (11)	Gamified SmartCAT, Triumf, HospiAvontuur, NM Francillette et al. (2018), Healthy-e Minds, Haddy, Grow It! App, PuzzleWalk, Lumi Nova, Legend of Evelys, The Guardians
Curiosity (11)	REACH, The Loom, RegnaTales, Happify Breather, Triumf, Serenity, NM Fraiwan et al. (2015), Lumi Nova, NM Samonte et al. (2022), AquaSnap, The Guardians
Challenge (9)	EVO Project, Personal Zen, Relax and Race, The Loom, Gamified SmartCAT, Drug Defence, HospiAvontuur, Match Emoji, Haddy
Fantasy (9)	RegnaTales, Triumf, Drug Defence, Journey to the West, SPARX Japanese-adapted, Magis, MyCQ, AquaSnap, The Guardians
Self-assessment (9)	SupperBetter, PuzzleWalk, I Love Breast, POD Adventures, Triumf, Healthy-e Minds, Grow It! App, Gamified SmartCAT, The Guardians
Relatedness (7)	SupperBetter, Triumf, MindMax, NM Fraiwan et al. (2015), Grow It! App, PuzzleWalk, LINA
Competence (6)	Relax and Race, Gamified SmartCAT, Triumf, Grow It! App, PuzzleWalk, AquaSnap
Support (6)	REACH, Gamified SmartCAT, POD Adventures, Healthy-e Minds, SPARX Japanese-adapted, PuzzleWalk
Feedback (3)	Gamified SmartCAT, Match Emoji, Grow It! App

### Study evaluations and main results

For 39 out of the 40 Serious Games, at least one article describes some type of evaluation, while some games have multiple articles describing (different) evaluations (for some Serious Games, several articles were extracted). For one Serious Game, no evaluation is reported (NM Fraiwan et al. 2015 [63]). Out of the articles that report an evaluation, five articles describe ongoing studies or protocols [45, 64–67] while the remaining 37 articles describe completed evaluations.

#### Socio-demographics characteristics

For those articles that describe completed evaluations, the sample population is diverse. Specifically, adults were involved in 21 studies, while minors were included in 18 studies. 22 of the studies recruited participants from a clinical population.

#### Study design

Regarding the study design, 12 Serious Games were rigorously evaluated by Randomized Controlled Trials (RCTs) or pilot RCTs (i.e., Relax and Race, The Loom [68], Happify Breather [69], Raw Hand [70], PuzzleWalk [71], I Love Breast [72], Drug Defence [73], POD Adventures [74], NM Vara et al. 2022 [75], LINA [51], ACE, and EVO Project [76] (see Section 3.11.4 for details). The remainder of the evaluations performed are non-randomized controlled trials, and prospective or quasi-experimental studies.

#### Evaluation target and measurement instruments

Eighteen articles focused on the effects on the clinical symptomatology of the participants, while 38 evaluated factors related to the user experience such as feasibility, acceptability, usability, opinion, satisfaction, or engagement. Only four measured game factors such as playability or game experience.

A wide variety of standardized psychological questionnaires, such as Beck Depression Inventory-II (BDI-II) [77] and National Institute on Alcohol Abuse and Alcoholism (NIAAA) Alcohol Screening Test [78], are the most widely used instruments to measure the effect on symptomatology. Bio-measures were used to a lesser extent (for example, Serenity [46] uses heart rate and oxygen levels to measure stress, Happify Breather [69] uses heart rate variability to measure stress). In order to assess engagement, acceptability and feasibility, passive data collected by the Serious Game-supported treatment, such as the duration of use (e.g., I Love Breast [72]) or amount of accesses (e.g., Gamified SmartCAT [79]) to the application are collected. A large majority of articles focusing on user experience evaluation are based on self-developed questionnaires or qualitative questions (e.g., RegnaTales [80], Triumf [81], among others). The use of standardized questionnaires, such as the System Usability Scale (SUS) [82], is less common (Legend of Evelys [83] and Gamified SmartCAT [84]). Qualitative interviews are also employed in order to extract qualitative feedback on user experience and satisfaction (e.g., in NM Francillette et al. (2018) [85], I Love Breast [72], among others). Regarding the instruments to evaluate game-related measures such as playability or game experience, the `Fun Toolkit' [86] (i.e., in RegnaTales [80]) and standardized questionnaires, such as the Game Experience Questionnaire (GEQ) [87] (i.e., in JIB [88]), the Game User Experience Satisfaction Scale (GUESS-18) [89] (i.e., in LINA [51]), and the GAMEFULQUEST [90] (i.e., in Triumf [67]), were used.

#### RCTs and pilot RCTs: main findings

While a meta-analysis was not judged as suitable due to the emerging nature of this area, and the wide variety of intervention targets and formats, the RCT's within the corpus were examined with respect to the reported outcomes. The principal findings of evaluation studies suggest that Serious Games could be a useful tool for helping people with mental health conditions or symptoms. The majority of RCT and pilot RCT studies aimed at effect and reported outcomes in which participants using a Serious Game show an improvement in the targeted symptoms. Specifically, the results indicate significant reductions in stress [68], and preliminary evidence for the efficacy to improve physiological stress recovery [69] and OCD symptoms [70]. Studies also report longer usage time in the Serious game-based treatment compared to the control group condition and, therefore, more engagement [71–74]. Moreover, study results suggest that Serious Games could provide an effective tool for teaching both emotion regulation techniques [75] and long-term knowledge about the effects of alcohol [73]. On the other hand, in some cases, no significant differences in symptoms compared to the control group were reported [51, 71, 72].

Additionally, those studies that report satisfaction and engagement indicate better results in the Serious Game use condition compared to the control conditions (i.e. receiving conventional treatment; being allocated to a waiting list; using a commercial app; watching a video documentary; receiving an informative digital flyer). The studies also support the feasibility and acceptance of this type of treatment delivery. In individuals with breast cancer, serious gaming has been reported to result in greater treatment satisfaction, improved medication adherence, lower incidence of side effects, and better quality of life, although no differences in anxiety and depression were reported compared to the control group [72]. Another result obtained is the possibility of recruiting more people in less time and at a lower cost to conduct an RCT study with a Serious game condition [73].

The details of these RCT and pilot RCT studies, including the name of the game, psychological approach and target, study sample size, experimental condition and main findings, are reported in Appendix Section 6.7.

## Discussion

The extracted data presented in Section 3 gives an overview of the use of smartphone-based Serious Games to address mental health problems. In this section, we will discuss the results with the aim of answering the established research questions, identifying gaps in the research field, and highlighting future research directions.

### Volume of research

The corpus of this research revealed a significant rise in the interest in using Serious Games for mental health care. Whereas the field could be considered as emerging between 2014 and 2021, in 2022 we detected a substantial number of research outputs. It remains to be seen if the increased interest is sustained in the future.

### Matching game genres to therapeutic elements

Our work highlights a dominance of action and arcade games (compare Section 3.1). An advantage of this genre can be found in its affordability: they are affordable to develop—as they do not require a lot of content or complex elements—and also to use in a psychological intervention context—as they can be completed in a short period of time. Still, overall we found a diverse range of game genres. Amongst the genres, a wide range of therapeutic elements were utilized to improve mental health. Puzzle games in particular seem to be suitable for incorporating psychological techniques such as cognitive restructuring or problem-solving strategies. The adventure and role-playing genres may be particularly suitable for interventions that target emotion management, social and communication training, and empathy training. Simulation games, on the other hand, especially when delivered in virtual reality, were generally used to practice relaxation techniques, and coping strategies, and also for exposure. The corpus considered in this paper contained few strategy games, even though they could be suitable for practising cognitive skills, long-term planning and decision-making. It is also worth mentioning that there is a notable absence of sports and shooter games. A reason might be that these genres are often focused on competitive and violent games, which may not be perceived as suitable for people with mental health problems.

### Game design process

We saw in Section 3.2 that for almost half of the games, no details regarding the design process were reported. For the remainder, participatory design is the most commonly employed approach. This design process, in conjunction with the iterative and user-centered approach [91], offers various advantages for the design of mental health games, particularly, the ability to facilitate collaboration and gather continuous feedback from both end-users and mental health experts. Such approaches help to make the game relevant and effective in fulfilling its intended purpose while ensuring usability and acceptability for the users. The use of different approaches in combination suggests that there is no single established approach to carry out this process yet. It is noteworthy that among the papers reporting the design process, a vast majority collaborated in interdisciplinary teams, including different domain experts and users. It has been noted that some Serious Games have been designed by directly transferring an existing psychological program or protocol. This strategy benefits from the use of already established knowledge and techniques and can be argued to increase the chances of the success of a Serious Game. The role of game-based delivery in such cases is to promote the attractiveness of the psychological program in order to help with treatment adherence, a problem faced by internet interventions [92]. However, it should be noted that such a direct translation of the psychological program into a game format may not always be possible or appropriate, and should in any case always be re-assessed and validated in the new format. Moreover, by translating existing protocols directly into the digital space, opportunities that technology offers might be overseen. Therefore, not only psychological protocols but also technical capabilities and further game elements (that e.g., could increase adherence) should be considered during the design process.

### Opportunities for increased use of smartphone capabilities

Smartphones have a wide range of features to host a variety of content, offer advanced functionalities (e.g., context awareness [93], biofeedback [94]), and hence could provide both richer and more tailored experiences. They are affordable, ubiquitous, and unobtrusive. Serious games can take advantage of these features to enhance both the gaming experience and to meet the needs of psychological treatments [21]. Still, we found that only a minority of Serious Games introduce capabilities beyond the display of multimedia content (see Section 3.3). While individual papers reported the use of accelerometers (e.g., to promote physical activity) or GPS and cameras (e.g., for exposure or bio-feedback), overall the smartphone’s interaction capabilities could be further exploited, such as vibrations (e.g., for concentration in meditation or controlling breathing rhythm in relaxation exercises). A reason behind the scarcity of the use of these features may be the added difficulty of reliably integrating and interpreting sensor measurements into an effective video game, the lack of awareness of developers about the potential of sensors in mental health care, or concerns about the privacy implications [95].

### Role of Serious Games within psychological care

In this corpus, about 3/4 of the games were designed for treatment, and only about 20\% for prevention, and about 5\% for assessment (see Section 3.4). We observed that many games focusing on the prevention of mental health problems aim to generally improve mental well-being and do not necessarily target specific mental health diagnoses. Exploiting their immersive nature and real-time, in-situ interactions with the patient, prevention-focused Serious Games have the potential to reduce the burden of mental health problems, by developing and/or fostering skills and capabilities to prevent or halt emerging mental health problems (primary and secondary prevention). Prevention is of particular interest in the current post-COVID-19 era, where there is an increase in emotion-related problems and increased stress on national health systems [1, 96]. In relation to how Serious Games are used in mental health care (stand-alone or as a complement), we want to highlight that the vast majority (about 87\%) of games are used as a stand-alone psychological treatment. Additionally, over 90\% have been designed to be self-administered, without the need for complementary therapy (such as face-to-face sessions). Designing such therapies to be entirely self-administered could be risky, especially considering that the dropout rates are one of the weaknesses of technology-based interventions. Nevertheless, it has been demonstrated that entirely self-administered psychological interventions are effective [17], and the inclusion of human support in internet-based programs enhances their efficacy and adherence [28, 97].

Even though this research is still in the early stages, it implies a potential advancement in expanding the accessibility and reach of psychological treatments, even though they require caution regarding safety and clinical supervision [98].

### Psychological condition targeted

As we show in Section 3.6, a diversity of different psychological conditions and symptoms were targeted. Our corpus reveals various conditions that were targeted only by individual games, and even the most targeted psychological conditions were addressed in no more than five games. This indicates a need for further exploration of the possibilities of Serious Games and identifying novel treatment options. In line with the prevalence of mental disorders and their symptoms [1], we found the affective disorders and symptoms of stress, anxiety, and depression amongst the most addressed targets. It is however surprising that the number of Serious Games addressing depression is relatively low, compared to the abundance of mHealth apps which address this target [20]. Interestingly, even though among the first mental health mobile applications were those intended to treat specific phobias [99], no such Serious Games were found that met the inclusion criteria (EC6—no applications explicitly developed for or tested on smartphones). The most addressed psychological target was not a clinical condition or symptom, but rather prevention and promotion of well-being. This corresponds with evolving psychological insights; whereby mental health is considered not only to be the absence of mental health problems but also the implication of a positive emotional state and the ability to adapt [100]. As such, psychological treatments increasingly promote addressing positive affect, well-being, and resilience [101], which can help to cope with challenging situations and boost protection against mental health problems [102, 103].

### Building on psychological frameworks

Even though all games in the corpus refer to psychological strategies (see Section 4.8), about 2/3 of the articles do not specify an underlying psychological framework. Amongst the remainder, the most followed framework is CBT, which is considered to be one of the most significant developments in the treatment of psychopathology over the past five decades [104]. CBT approaches are both widely used and widely researched, with extensive scientific evidence supporting the efficacy of CBT in the treatment of a variety of mental disorders [105, 106]. As a structured and short-term therapy in which patients develop more adaptive thoughts and behaviors [107], CBT is one of the frameworks that is well suited for Serious Games use via smartphones since its structure can be integrated into the form of game quests or levels and players can reflect about their beliefs through the gameplay in an interactive way.

However, it is important to remark that some games introduced third-wave psychotherapy techniques, such as ACT and PP strategies. These strategies prioritize the holistic promotion of psychological and behavioral processes associated with health and well-being over the reduction or elimination of psychological and emotional symptoms [108]. PP strategies also function to enhance well-being, rather than to reduce psychopathological symptoms [109]. However, only a small number of games utilise these strategies. Considering that our corpus revealed that prevention and promotion of well-being is an important target of serious games (Section 3.6), we see more potential for the use of these strategies.

### Psychological strategies

As reported in Section 3.8, a wide range of psychological strategies were employed. The predominant technique in this corpus was relaxation, followed by psychoeducation and emotion management. Relaxation is defined as a physiological state contrary to anxiety and other negative emotional states [110]. For this reason, relaxation is a widely accepted and recommended technique in clinical practice which can be helpful to regulate and manage negative emotions at the moment of feeling them [111]. Relaxation techniques are well suited for Serious Games, as their concrete and simple instructions are easy to incorporate into a game format. Game elements, such as graphics and sound, contribute to creating calming experiences through the immersion in a quiet and relaxing atmosphere. The integration of graphics and sound in Serious Games not only enhances the overall gaming experience but also magnifies the therapeutic advantages, helping in stress and anxiety reduction [112, 113]. Thus, it stands as an important aspect to consider in the development of games designed for promoting relaxation. Psychoeducation entails the education of the patient regarding their mental health status and the corresponding treatment. It constitutes a foundational component within various evidence-based interventions, and can also be easily integrated into serious games, for example as part of the conversation with characters, feedback provided by the game or as a resource in the game (e.g., an in-game book). Emotion management implies the detection, understanding, and effective management of current emotions, which in turn impacts an individual's psychological well-being [114] and social relationships [115]. Games can serve as a valuable tool for addressing emotional regulation, as they can evoke various emotions and facilitate their exploration through the game story, character interactions, and aesthetics (graphics and sound). With respect to how psychological techniques are mapped onto game elements, we showed that gameplay is the most used to incorporate psychological content. This implies that the challenges encountered and actions performed by the player are consistent with the application of psychological techniques. By embedding these techniques into a game as an integral part of gameplay, the aim is to make the therapeutic process more attractive to the patients, so that required skills can be acquired and practiced in a way that feels fun and challenging. This approach has the potential to increase the patient’s motivation and engagement and may ultimately lead to more effective therapeutic outcomes [32]. Other than that, graphics and stories are commonly used to represent psychological elements. Using graphics can be helpful to illustrate concepts related to psychological content, serve as visual cues and reinforcement of applied techniques or help to create a certain mindset beneficial for the intervention. Stories are often employed as a vehicle to convey the psychological message, while gradually revealing the story can also be a way to motivate the player to continue playing, and hence continue the treatment [36]. For other established game elements, we found only sporadic use.

### Engagement and motivation

Serious games and gamification have the potential to improve the impact of online treatment outcomes [36]. Consistent with the motivation to apply Serious Games in order to engage users [32], the review has identified various engagement techniques aimed at reducing the dropout rate of self-administered treatments (see Section 3.10). Dropout rates are known to be high in internet-based treatments in general (varying from 2\% and 83\% with a weighted average of 31\% [116]), and smartphone-based treatments in particular. Illustratively, dropout rates up to 47.8\% have been reported for smartphone apps for depressive symptoms [117]. This Scoping Review reveals that the most commonly used engagement mechanism is rewards, whether tangible (e.g., toys, pens, stickers) or within the game (e.g., points, coins), despite the fact that they have negative connotations in the literature (negative effect on the user's intrinsic motivation [118], use of points and scores as a common criticism of gamification [119]). Reasons might include easy implementation, as well as short-term encouragement for the user to continue playing, which is important to prevent immediate dropout. Besides extrinsic motivation through rewards, it is also common for Serious Games to incorporate factors that influence intrinsic motivation (e.g., offering autonomy, promoting curiosity, presenting challenges, or creating relatedness). Providing feedback might also be crucial in maintaining players' motivation and supporting positive clinical outcomes, as the literature shows the potential benefits of clinical feedback in mental health [120]. The inherent characteristics of games make them suitable for incorporating a range of factors to promote intrinsic motivation, as they support open, responsive, imaginative, and safe worlds where bonds and social networks can be formed with characters and other players.

### Demographics of study participants

About half the studies included in this corpus involved participants who were minors; thus young people are considered as a key target group for treatments delivered via games. While this can be partially explained by the potential appeal of games to younger people, research suggests that about 50\% of mental health disorders that occur in adulthood begin before the age of 15, and 75\% begin before the age of 18 [121]. Thus, it is important to design treatments that can be offered to broad age groups, including children and adolescents.

### Reported study outcomes

As detailed in Section 3.11.3, 88\% of studies focused on evaluating user experience, while only 41\% studied the effect on psychological symptomatology. This may partly be due to the infancy of the field, whereby the use, acceptability and user experience of Serious Games are explored in order to further improve them before focusing on larger-scale studies which measure the effect on symptoms. Nevertheless, approximately one-third of all games have been evaluated using RCT or pilot RCT methods (see Appendix Section 6.7 for details). The vast majority of these aimed to assess the effectiveness or effect, and have yielded promising results. In spite of the fact that, as observed in Section 3.6, serious games centered around affective disorders constitute slightly over one-third of the identified serious games, only one game specifically targeting depression has conducted an RCT. Several RCTs also report on studies in which higher levels of user adherence are observed in the Serious Game condition. This result suggests that Serious Games could be a promising solution to reduce dropout rates often seen in Internet-based interventions [92].

Regarding the evaluation measures employed in the studies, effects on symptomatology are mostly measured using standardized psychological questionnaires. A significant number used their own questionnaires and qualitative methods for user experience evaluation, allowing a tailored understanding of the gaming experience. While these methods are suitable for formative evaluations where the main aim is to gain insight and obtain a rich picture of user experience, such self-crafted studies are less appropriate for summative evaluations, and make it difficult to compare technology interventions in a systematic way. Additionally, even though the subject of evaluation is a game, there is a lack of use of gaming-specific measures. On a related note, while specific evaluation instruments for mobile applications in mental health are available [122], to the best of our knowledge there are no such instruments for serious games for (mental) health.

### Limitations

This Scoping Review is prone to the same limitations as all such review types. First, this Scoping Review included only articles published in international journals and full articles published in conference proceedings written in English. For this reason, potentially relevant studies published elsewhere may have been missed. Secondly, it is possible that certain articles may have been omitted from the analysis if the required information to assess their eligibility based on the inclusion criteria was not provided. Thirdly, even though the terms employed in the research query have been extracted from prior reviews and taking into account MeSH Terms, it is possible that the use of alternative terminology by authors caused relevant articles to remain undetected. Finally, the screening and data extraction process may be subject to inaccuracies, even though double, independent classification with a referee mechanism for disagreements was in place (see Section 2.4).

## Conclusions

This article presents a Scoping Review on the use of Serious Games for smartphones in the field of mental health, with particular attention for the technical and psychological dimensions. Our review resulted in 43 relevant studies describing a total of 40 different Serious Games. While we observe a rising interest in Serious Games for mental health via smartphones, this area of research is still in its nascent stages. Our review shows a wide variety of game genres addressing miscellaneous mental health problems, exploiting smartphone features to various extents and applying different psychological strategies. Promising positive results towards effect and patient engagement were shown, but further research is needed to consolidate and generalize results. In particular, based on this work, we make the following recommendations for future research:

1. More research reporting towards effective design processes is needed, particularly on how to successfully identify and integrate suitable psychological theories and practices into different aspects of the game, to achieve highly engaging and effective smartphone-based Serious Games for mental health care.
2. Further exploration of the use of advanced smartphone features (e.g., anywhere/anytime nature, sensors, peripheral hardware) to be integrated into Serious Games to enhance entertainment value, user engagement and effectiveness of treatment.
3. Despite the understandable focus on interventions, there is room for further research towards smartphone-based Serious Games as prevention, assessment, follow-up or relapse prevention tools.
4. There is a need for the development of standardized and/or equivalent measurement instruments for user experience and game-specific aspects, in order to achieve more consistent and comparable evaluations. Particularly, understanding the effectiveness of engagement mechanisms is crucial to address high attrition rates and low adherence.
5. As the field matures, there is a need for more rigorous RCT studies towards the effectiveness of smartphone-based Serious Games for mental health care, in order to further build trust and foment acceptance of this type of treatment.

Patients and practitioners stand to benefit from new types of therapies, such as those offered by Serious Games, as they have the potential to help lower the barriers to access to treatment, may better suit certain audiences and circumstances, and help to reduce the treatment gap. Nevertheless, Serious Games still mainly reside in the research realm, and few commercial-grade clinically proven serious game-based interventions are available. Therefore, we call upon practitioners to help evolve this research field by actively engaging in the design (i.e., through participatory, iterative and/or user-centred design processes) and evaluation (i.e., through rigorous evaluations, from case studies to RCTs) of Serious Games for mental health. Strong cooperation between researchers, game developers and practitioners is key to strengthening scientific evidence of Serious Games in mental health care, understanding critical success factors, and eventually, acceptance and uptake of Serious Games in general practice.

## Appendices

### Search strings

#### General search query

(Smartphone OR mHealth OR mobile OR "mobile-based" OR "smartphone-based") \\ AND ("Video Games" OR game OR gamif*)

AND ("Mental Health" OR "Mental Disorders" OR Psychotherapy OR "Depression" OR "Stress, Psychological" OR "Affect" OR "Anxiety" OR "mental disease" OR "mental illness" OR "psychotherapy" OR depress* OR distress OR wellbeing OR "well-being" OR resilience OR psycho^‡^ OR cognitive behavioral.^‡)^

#### PubMed search query

((Smartphone[Title/Abstract]) OR (mHealth[Title/Abstract]) OR (mobile[Title/Abstract]) OR ("mobile-based"[Title/Abstract]) OR ("smartphone-based"[Title/Abstract])) AND (("Video Games"[Title/Abstract]) OR (game[Title/Abstract]) OR (gamif*[Title/Abstract])) AND (("Mental Health"[Title/Abstract]) OR ("Mental Disorders"[Title/Abstract]) OR (Psychotherapy[Title/Abstract]) OR ("Depression"[Title/Abstract]) OR ("Stress, Psychological"[Title/Abstract]) OR ("Affect"[Title/Abstract]) OR ("Anxiety"[Title/Abstract]) OR ("mental disease"[Title/Abstract]) OR ("mental illness"[Title/Abstract])OR (depress*[Title/Abstract]) OR (distress[Title/Abstract]) OR (wellbeing[Title/Abstract]) OR ("well-being"[Title/Abstract]) OR (resilience[Title/Abstract]) OR (psycho[Title/Abstract^1^] OR (cognitive behavioral[Title/Abstract]^‡^)).

#### PsycInfo search query

Note: on December 2021, searching in title and abstract needed to be done separately. Therefore, the next process was conducted:

**Table:** 

Step	Query	ID Search
1	TI Smartphone OR TI mHealth OR TI mobile OR TI "Mobile-based" OR TI "Smartphone-based"	S1
2	AB Smartphone OR AB mHealth OR AB mobile OR AB "Mobile-based" OR AB "Smartphone-based"	S2
3	TI "Video Games" OR TI game OR TI gamif*	S3
4	AB "Video Games" OR AB game OR AB gamif*	S4
5	TI "Mental Health" OR TI "Mental Disorders" OR TI Psychotherapy OR TI Depression OR TI "Stress, Psychological" OR TI Affect OR TI Anxiety OR TI ( "mental disease" OR "mental illness") OR TI depress* OR TI distress OR TI ( wellbeing OR "well-being") OR TI resilience	S5
6	AB "Mental Health" OR AB "Mental Disorders" OR AB Psychotherapy OR AB Depression OR AB "Stress, Psychological" OR AB Affect OR AB Anxiety OR AB ( "mental disease" OR "mental illness") OR AB depress* OR AB distress OR AB ( wellbeing OR "well-being") OR AB resilience	S6
7	S1 OR S2	S7
8	S3 OR S4	S8
9	S5 OR S6	S9
10	S7 AND S8 AND S9	S10

On January 9th of 2023, the following search query was conducted (now supporting searching in title and abstract in one query):

(title(mHealth) OR title(mobile) OR title(Smartphone) OR title("Mobile-based") OR title("Smartphone-based") OR abstract(mHealth) OR abstract(mobile) OR abstract(Smartphone) OR abstract("Mobile-based") OR abstract("Smartphone-based"))AND(title("Video Games") OR title(game)OR title(gamif*) OR abstract("Video Games") OR abstract(game) OR abstract("gamif*")) AND (title("Mental Health") OR title("Mental Disorders") OR title(Psychotherapy) OR title(depression) OR title("Stress, Psychological") OR title(Affect) OR title(Anxiety) OR title("mental disease") OR title("mental illness") OR title(depress*) OR title(distress) OR title(wellbeing) OR title("well-being") OR title(resilience) OR title(psycho^‡^) OR title(cognitive behavioral^‡^) OR abstract("Mental Health") OR abstract("Mental Disorders") OR abstract(Psychotherapy) OR abstract(depression) OR abstract("Stress, Psychological") OR abstract(Affect) OR abstract(Anxiety) OR abstract("mental disease") OR abstract("mental illness") OR abstract(depress*) OR abstract(distress) OR abstract(wellbeing) OR abstract("well-being") OR abstract(resilience) OR abstract(psycho^‡^) OR abstract(cognitive behavioral^‡^)).

#### ACM Guide to computing literature search query

(Title:(Smartphone OR mHealth OR mobile OR "Mobile-based" OR "Smartphone-based") OR Abstract:(Smartphone OR mHealth OR mobile OR "Mobile-based" OR "Smartphone-based")) AND (Title:("Video Games" OR game OR gamif*) OR Abstract:("Video Games" OR game OR gamif*)) AND (Title:("Mental Health" OR "Mental Disorders" OR Psychotherapy OR Depression OR "Stress, Psychological" OR Affect OR Anxiety OR "mental disease" OR "mental illness" OR depress* OR distress OR wellbeing OR "well-being" OR resilience OR psycho^‡^ OR cognitive behavioral^‡^) OR Abstract:("Mental Health" OR "Mental Disorders" OR Psychotherapy OR Depression OR "Stress, Psychological" OR Affect OR Anxiety OR "mental disease" OR "mental illness" OR depress* OR distress OR wellbeing OR "well-being" OR resilience OR pyscho^‡^ OR cognitive behavioral^‡^)).

###  Serious Games and corresponding studies

**Table:** 

Serious Game name	Article(s)
ACE	[76]
AquaSnap	[123]
Drug Defense	[73]
EVO Project	[76]
Gamified SmartCAT	[79, 84]
Grow It! App	[124]
Haddy	[52]
HAPPIA	[125]
Happify Breather	[69]
Healthy-e Minds	[45]
HospiAvontuur	[48]
I Love Breast	[72]
JIB	[88]
Journey to the west	[65]
Legend of Evelys	[83]
LINA	[51]
Lumi Nova	[126]
Magis	[127]
Match emoji	[47]
MindMax	[128, 129]
MyCQ	[123]
NM Vara, et al. (2016)	[75]
NM^§^ Francillette, et al. (2018)	[85]
NM^§^ Fraiwan, et al. (2015)	[63]
NM^§^ Samonte, et al. (2022)	[112]
No Fume	[50]
Personal Zen	[113]
POD Adventures	[64, 74, 130, 131]
PuzzleWalk	[71]
Raw Hand	[70]
REACH	[132]
RegnaTales	[80]
Relax and Race	[68]
Serenity	[46]
SmokeFreeBrain	[133]
SPARX (Japanese-adapted)	[66]
SupperBetter	[134]
The Guardians	[135]
The Loom	[68]
Triumf	[67, 81]

NM stand for “Not Mentioned”

###  Serious Game names and generes

**Table:** 

Serious Game name	Description	Genre
ACE	ACE is a set of mini-games to assess cognitive ability	Action and arcade
AquaSnap	AquaSnap is a cognitive-enhancing video game that focuses on the five cognitive domains measured by MyCQ. Players assume the role of a submarine explorer, venturing into the depths of the ocean to capture captivating fish photographs and accomplish missions. By gaining experience and currency through successful photography and mission completion, players unlock new areas to explore	Action and arcade
Drug Defense	In this game focused on alcohol effects on the liver, players defending the liver by placing in turrets while shooting enzymes. Through reading the character's journal entries, players immersed themselves in the story of Miguel, a college student who faced relatable challenges like loneliness, heartbreak, and academic failures. As Miguel's excessive alcohol consumption escalated, the game highlighted the importance of support from friends and family in helping him overcome his struggles	Strategy
EVO Project	This mobile immersive video game builds upon the cognitive intervention NeuroRacer, where players control a character navigating an infinite environment, evading obstacles. It aims to assess and improve cognitive functioning	Action and arcade
Gamified SmartCAT	This program consists of various modules that incorporate four mini-games to practice cognitive-behavioral therapy (CBT) skills for managing anxiety. The modules include therapist-guided sessions, interactive mini-games, relaxation techniques, and task assignments selected by therapists	Puzzle
Grow It! App	Designed for adolescents aged 12–25, the multiplayer serious game app utilizes the experience sampling method (ESM) to monitor their daily thoughts, behaviors, and emotions. By incorporating cognitive-behavioral therapy-based challenges, the app aims to enhance self-insight and promote adaptive coping strategies	Puzzle
Haddy	This app offers a diverse range of features, including four games (biofeedback, card, arcade, and memory) to engage users. It also provides two relaxation techniques, a breathing exercise, and yoga videos, promoting stress reduction and mindfulness. Additionally, the app incorporates positive messaging and a mood tracking calendar as two additional features	Action and arcade Puzzle
HAPPIA	NM	NM
Happify Breather	In this calming game, players are immersed in serene nature scenes such as underwater coral beds, tropical beaches, and mountaintops. As players continue to breathe deeply and regularly, they automatically traverse through the environment. With each breath, the scenes become more complex and beautiful, showcasing the blossoming of coral polyps and the growth of vibrant flowers, creating a soothing and visually captivating experience	Action and arcade
Healthy-e Minds	The smartphone app is designed to support individuals with diagnosed severe mental illness (SMI) throughout their treatment journey. It monitors health measures, provides interactive questionnaires, and delivers educational and motivational multimedia content. As a component of this app, "Friendly Farmers" serves as a farming simulation game where players earn in-game currency by fulfilling customer goals, adding an engaging and rewarding element to the overall experience	Simulation
HospiAvontuur	Designed for the final days before a hospital admission, this game immerses players in various surgical procedure scenarios using an avatar. Each scenario, such as being at home, heading towards the hospital, or being at the hospital, presents different tasks related to patient preparations. For instance, players must prepare a suitcase at home, finding and collecting the correct items while learning what is allowed to be taken to the hospital. This interactive experience aims to educate and familiarize players with the necessary steps before undergoing a surgical procedure	Adventure
I Love Breast	This personalized game features an avatar based on the player's medical information. Players simulate a healthy routine by engaging in activities such as visiting their home, hospital, pharmacy, gym, and cooking nutritious meals. Completing quests, such as taking medication on time or going for a walk, earns heart coins for avatar customization	Simulation
JIB	JIB is a game designed to address alcoholism by allowing players to experience the consequences of alcohol abuse within a forest-themed narrative. The game portrays the effects of alcohol through increased game speed, creating an illusion of heightened enjoyment, but also illustrating the potential for premature death. Players control an indigenous hero at the bottom of the screen, avoiding encounters with jaguars and snakes while collecting drinks to increase points and game speed, symbolizing the similarity to consuming alcohol	Action and arcade
Journey to the west	This game aims to address stress and depression by incorporating cultural factors and utilizing the Salutogenesis framework, which emphasizes health and well-being rather than only treat disease. The core mechanic of "breath" plays a significant role in combat and world interaction, where players learn proper breathing techniques to connect their inner and outer worlds. By applying these techniques, players can work "magic" and visually transform the landscape, reminiscent of the acclaimed game Okami's Celestial Brush mechanics. This integration of cultural elements and breath mechanics enhances the gameplay experience while promoting mental well-being	Role-play
Legend of Evelys	This video game depicts a fantasy storyline where citizens in a fictional world experience negative emotional states mirroring the impact of the COVID-19 pandemic and its associated mental health challenges. It offers players an immersive experience that reflects and explores these difficult emotions within a fictional context	Role-play
LINA	LINA is an immersive and interactive shared narrative where players collaborate using augmented reality (AR) to uncover artifacts left by a fictional classmate. Together, they work to determine the whereabouts and reasons behind her disappearance, engaging in a collective effort to solve the mystery	Adventure
Lumi Nova	In this intergalactic role-playing adventure, players embark on a journey as treasure hunters to save the galaxy and explore the vast universe. Along the way, they assist characters on different planets while simultaneously undergoing training to overcome real-world fears	Role-play
Magis	A Magical Adventure is a mobile game that promotes psychological flexibility through its dialogue and plot. Players engage in Acceptance and Commitment Therapy (ACT)-based conversations with various characters in the game world. These interactions help players process challenging thoughts and emotions, identify personal values, take actions aligned with their values, and develop empathy by considering others' perspectives. By enhancing psychological flexibility and related skills, the game aims to improve players' overall well-being	Adventure
Match emoji	Match Emoji is a game where the objective is to match emojis of similar colors together, earning points and advancing through the game. The game features six different colored and shaped emojis that represent digital expressions of emotions, ideas, and personality	Puzzle
MindMax	MindMax consists of three primary components: psychoeducational modules for wellbeing training, a social community feed, and a casual game called Flick Footy. The app incorporates gamification elements where playing Flick Footy requires in-app currency called "footies," which can be earned by completing training modules and engaging with other users through posting or commenting in the community feed	Action and arcade
MyCQ	The MyCQ app is designed to assess cognitive functioning across five domains: attention, processing speed, working memory, episodic memory, and executive function. By evaluating performance in these cognitive areas, the app provides users with insights into their cognitive abilities and helps identify areas for treatment through AquaSnap	Action and arcade
NM Vara, et al. (2016)	The app features a feather that continuously moves up and down on the device screen for a total of 45 s, with each cycle lasting 2.5 s. The purpose of this visual cue is to guide users to synchronize their breathing with the feather's movement, encouraging them to inhale and exhale at the same rhythm	Action and arcade
NM Francillette, et al. (2018)	In this game, players navigate the ocean depths in a submarine, collecting aquatic creatures that are converted into tokens, the game's currency. The density of creatures decreases with each collection, but players can positively impact the creature density by spending tokens. To earn more tokens, players must engage in physical activity, and the number of tokens obtained is proportional to the difficulty level of the exercise, which requires more time to complete	Action and arcade
NM Fraiwan, et al. (2015)	The game follows the story of Sami, a five-year-old boy leading a typical life with his family. Starting from his morning routine and continuing throughout his day at school, on the school bus, and at the playground, Sami encounters various situations and events. The objective is to assist Sami in collecting as many toys as possible to fill his new closet, which his father provided. By making appropriate decisions in different circumstances, Sami can earn rewards and gifts. The game aims to visually prepare children for challenging daily situations by providing a practical and visual experience	Adventure
NM Samonte, et al. (2022)	The psychotherapeutic virtual reality system offers users the opportunity to explore popular relaxation spots as a tourist or local traveler. The game mechanics involve walking by tilting the head in the desired direction (300 to 450 degrees), interacting with objects by holding the reticle near them, and pausing the game by looking upwards to access the pause menu. The game is specifically designed to introduce various provincial and local destinations in the Philippines, such as Palawan, Sagada, and Pagudpod, as part of the therapeutic experience	Simulation
No Fume	No Fume is a game that combines a quiz on tobacco knowledge with four additional mini-games to emphasize the positive effects of a tobacco-free life and the negative consequences of tobacco use. The mini-games include the Bus game, showcasing the impact of tobacco on sports and health; the Environment game, highlighting the effects of tobacco on the environment; the Piggy Bank game, illustrating nicotine dependence and the financial implications of tobacco consumption; and the Accepting and Refusing Tobacco game, promoting resistance to peer pressure and the ability to say no to tobacco	Puzzle
Personal Zen	In this game, two animated characters appear on the screen and burrow into a hole. As one of the characters moves, a path of rustling grass is left behind. The player's task is to trace the path with their finger, starting from the burrow, as quickly and accurately as possible. The trail of grass always appears in the location of the non-threat character. The paths consist of separate "tufts" of grass, with the number of tufts randomly varying between five and eight. When a tuft is correctly traced, it illuminates to indicate successful tracing	Action and arcade
POD Adventures	POD consists of two sections. In the "Adventures" section, players engage in short vignette-based stories featuring diverse adolescent characters and their problem-solving journeys. Through interactive conversations, players actively participate in these journeys. The second section, "My POD," guides players through problem-solving steps for their own issues using a series of forced-choices and open-text questions	Adventure
PuzzleWalk	PuzzleWalk is a spot the difference puzzle game featuring 660 major city images from around the world. A unique aspect of the game is its conversion algorithm, which directly converts the user's accumulated steps into game-solving time, thereby encouraging physical activity (PA) participation. The game includes a gamified leaderboard that ranks active users based on their steps and puzzle scores. At the end of each month, tangible rewards, such as US \$10 e-gift cards, are provided to the top three score leaders	Puzzle
Raw Hand	Raw Hand presents a collection of five mini-games designed to address specific challenges related to obsessive–compulsive disorder (OCD). These mini-games include contamination/washing, doubting/checking, symmetry/ordering, numbering/counting, and unacceptable thought/mental ritual. Each mini-game requires the player to engage in activities that demand selective attention, followed by a request to pause before completing the final task. For instance, players may be asked to touch pictorial germs to remove them and then wait for a specified time (e.g., five seconds) before removing the last one	Action and arcade
REACH	REACH is a mHealth platform consisting of six modules, including S.T.I.C. activity, S.T.O.P. activity, Daily Diary, Worryheads game, and relaxation. The Worryheads game focuses on addressing fears and related thoughts. Players are presented with statements about their fears (as part of S.T.O.P activity) and must select the correct alternative thought from four options provided	Puzzle
RegnaTales	RegnaTales is set in a fantasy world where a balance exists between good and bad energy. However, the evil dark lord has disrupted this balance by tilting it towards negative energies. The player's objective is to restore the energy balance by completing seven missions that focus on social problem-solving skills	Role-play
Relax and Race	In the game Relax and Race, players control a small green dragon in a race where their velocity is determined by their level of relaxation, measured through their skin conductance. As the player relaxes, the dragon flies higher and faster	Action and arcade
Serenity	The application incorporates serene and harmonious Philippine-based geographical scenerios and locations, offering users a tranquil experience in a virtual world. The system's design draws inspiration from role-playing games, where players assume the role of a traveler exploring vacation spots in different settings, including day and night environments	Simulation
SmokeFreeBrain	SmokerFreeBrain is an app comprising three mini-games: "Crash the cigarettes," "Blow the balloon," and "Physical Exercise." In the first one, users aim to break as many cigarettes as they can within a minute. The second game tasks players with inflating a balloon by using the smartphone's microphone when it turns green. The final mini-game involves following a set of physical exercises demonstrated in a video	Action and arcade
SPARX (Japanese-adapted)	SPARX is an interactive fantasy game that guides users through seven modules, teaching essential cognitive-behavioral therapy (CBT) strategies. Upon starting the game, users meet the character of the Guide, who introduces them to the game world and its challenges. After selecting their avatar, users embark on the quest. At the end of each module, the avatar returns to the Guide, who explains how the learned "game skills" can be applied in real-life situations	Adventure
SupperBetter	SupperBetter employs a unique approach by reframing health-related factors as either enemies (negative influences) or power-ups (positive influences). The game also allows players to invite supportive individuals as allies who can send encouraging messages. Players are required to report these factors within the game, and they can be rewarded for their engagement	Puzzle
The Guardians	The Guardians is a pet-collection strategy game where players obtain pets and experience points through real-world activities. By leveling up their pets, players can embark on various missions to liberate the guardians of each realm who have been captured by enemies	Strategy
The Loom	In The Loom, the objective is to transform a winter scene into a summer scene. The speed of this transition is directly influenced by the player's level of relaxation. The more relaxed the player is, the faster the transformation from winter to summer occurs. This gameplay mechanic encourages players to focus on relaxation techniques and achieve a calm state of mind	Action and arcade
Triumf	The intervention includes various mini-games, including those that require the application of in-game learned information and cognitive challenges. It also incorporates entertainment games designed to offer cognitive distraction. The storyline revolves around saving Triumfland City by discovering and harnessing inner superpowers to tame the Disease Monster. The game includes monitoring features that assess the player's mental state through questions. Additionally, one of the mini-games focuses on recognizing the emotional states of the children in the city. Tic-tac-toe and other games are incorporated to provide cognitive distraction for the player	Adventure

###  Serious Games design process and people involved

**Table:** 

Serious Game name	Design approach	People involved
Gamified SmartCAT	Iterative User-centered	Mental health professionals Developers End-users (minors)
Grow It! App	User-entered Participatory design	Child and adolescent psychiatrist Developmental and clinical psychologist Analysts Game designers End-users (minors)
Haddy	User-centered Participatory design	Mental health professionals End-users (university students with anxiety
HAPPIA	Participatory design	Mental health professionals End-users
Healtyh-e Minds	NM	General health professionals Researchers
HospiAvontuur	Participatory design	Mental health professionals General health professionals Researchers Developers Educational specialists End-users (minor patients and parents)
Legend of Evelys	Participatory design	Mental health professionals
LINA	Iterative Participatory design	Mental health professionals Playwright Software developers Artists Adults with a parent with mental illness End-users (minors)
Lumi Nova	User-centered Participatory design	Clinical pracitioners Academics Game industry experts Teachers End-users (children and parents)
Magis	Prototyping	Mental health professionals
Match Emoji	Iterative	Mental health professionals Developers Educational specialists End-users (minors)
MindMax	Iterative Participatory design	Researchers Digital development specialists End-users
NM Samonte, et al. (2022)	Participatory design	Mental health professionals End-users (college students)
No Fume	Translation	NM
POD Adventures	Iterative User-centered Participatory design	Mental health professionals Developers Educational specialists End-users (minors)
Raw Hand	Theory-based	Mental health professionals Developers End-users (patients)
REACH	Translation Iterative User-centered Participatory design	Mental health professionals Developers Designers
RegnaTales	Translation	NM
SPARX (Japanese-adapted)	Translation	NM
SupperBetter	Translation	NM
The Guardians	Participatory design	NM
Triumf	User-centered Participatory design	General health professionals End-users (minor patients and parents)

###  Overview of the Serious Game psychological point of view

**Table:** 

Dimension	Category and Serious Games
Purpose of psychological care	**Prevention (9):** Grow It, App!, Journey to the West, LINA, Match Emoji, Magis, MindMax, NM Francillete et al. 2018, POD Adventures, REACH **Intervention (31):** AquaSnap, Drug Defence, EVO Project, Gamified SmartCAT, Haddy, HAPPIA, Happify Breather, Healthy e-Minds, HospiAvontuur, I Love Breast, JIB, Legend of Evelys, Lumi Nova, NM Samonte et al. 2022, NM Vara et al., NM Fraiwan et al., No Fume, Personal zen, POD Adventures, PuzzleWalk, Raw Hand, REACH, RegnaTales, Relax and Race, SmokereeBrain, Serenity, SPARX Japanese adapted, SupperBetter, The Guardians, The Loom, Triumf **Assessment (2):** MyCQ, ACE
Type of usage in psychological care	**Serious Game complete treatment (34):** ACE, AquaSnap, Drug Defense, EVO Project, Grow It! App, HAPPIA, Happify Breather, HospiAvontuur, I Love Breast, JIB, Journey to the West, Legend of Evelys, LINA, Lumi Nova, Magis, Match Emoji, MyCQ, NM Vara, et al. 2016, NM Francillette, et al. 2018, NM Fraiwan, et al. 2015, NM Samonte et al. (2022), No Fume, Personal Zen, POD Adventures, PuzzleWalk, Raw Hand, RegnaTales, Relax and Race, Serenity, SPARX Japanese-adapted, SupperBetter, The Guardians, The Loom, Triumf **Serious Game-supported treatment (6):** Gamified SmartCAT, Haddy, Healthy e-Minds, MindMax, REACH, SmokeFreeBrain **Complementary to treatment as usual (5):** AquaSnap, MyCQ, HAPPIA, Raw Hand, RegnaTales
Psychological target	**Prevention/Promotion well-being (8):** Happify Breather, Match Emoji, MindMax, The Guardians, POD Adventures, Grow It! App, LINA, Magis **Anxiety (6):** Gamified SmartCAT, Haddy, HAPPIA, Lumni Nova, Personal Zen, REACH **Substance Use/Abuse (6):** AquaSnap, Drug Defense, JIB, MyCQ, No Fume, SmokeFreeBrain **Stress (5):** Journey to the West, NM Samonte, et al. 2022, RegnaTales, Relax and Race, Serenity, The Loom **Emotional Disturbance due to medical procedure or illness (4):** HospiAvontuur, I Love Breast, Triumf, Legend of Evelys **Depression (4):** ACE, EVO Project, SPARX Japanese-adapted, SupperBetter **Severe mental health problems (2):** Healthy e-Minds, NM Francillete, et al. 2018 **Neurodevelopmental disorders (2):** NM Fraiwan, et al. 2015, PuzzleWalk **Emotion regulation (1):** NM Vara, et al. 2016 **Disruptive Behavior Disorder (1):** RegnaTales **OCD (1):** Raw Hand
Psychological framework	**CBT (12):** Drug Defence, Gamified SmartCAT, Grow It! App, Journey to the West, Legend of Evelys, Lumi Nova, HAPPIA, SPARX Japanese-adapted, Raw Hand, RegnaTales, Triumf **3rd Wave (2):** Magis, MindMax **PP (2):** MindMax, SupperBetter **Behavioral Activation (1):** The Guardians

###  Psychological strategies incorporated into the Serious Games supported treatments

**Table:** 

Psychological technique	Serious Game
Relaxation: 15	REACH, Relax and Race, The Loom, Gamified SmartCAT, POD Adventures, Happify Breather, Triumf, Serenity, NM Vara et al. 2016, Match Emoji, Journey to the West, SPARX Japanese-adapted, Haddy, NM Samonte et al. 2022
Psychoeducation: 11	REACH, I Love Breast, JIB, Triumf, Drug Defense, MindMax, Health-e Minds, SPARX Japanese-adapted, No Fume, Lumi Nova, LINA
Emotion management: 10	RegnaTales, Gamified SmartCAT, POD Adventures, Triumf, MindMax, Match Emoji, NM Fraiwan et al. 2015, Grow It! App, No Fume, Magis
Cognitive restructuring: 8	REACH, Gamified SmartCAT, Triumf, MindMax, Match Emoji, SPARX Japanese-adapted, Magis, Legend of Evelys
Exposure: 7	REACH, Raw Hand, Gamified SmartCAT, Triumf, HospiAvontuur, No Fume, Lumi Nova
Physical activity: 7	I Love Breast, Triumf, NM Francillette et al. 2018, Haddy, Grow It! App, The Guardians, SmokeFreeBrain
Coping strategies training: 6	RegnaTales, Gamified SmartCAT, Triumf, Journey to the West, Grow It! App, Legend of Evelys
Problem solving strategies: 6	RegnaTales, Gamified SmartCAT, POD Adventures, NM Fraiwan et al. 2015, SPARX Japanese-adapted, Grow It! App
Meditation: 6	Triumf, MindMax, SPARX Japanese-adapted, Legend of Evelys, RegnaTales, PODAdventures
Social and communication training: 5	REACH, RegnaTales, NM Fraiwan et al. 2015, SPARX Japanese-adapted, LINA
Positive psychology strategies: 5	SupperBetter, MindMax, Match Emoji, SPARX Japanese-adapted, SmokeFreeBrain
Behavioural activation: 4	I Lover Breast, SPARX Japanese-adapted, Legend of Evelys, The Guardians
Cognitive distraction: 3	Triumf, Match Emoji, Haddy
Motivation for treatment: 3	MindMax, Health-e Minds, LINA
Empathy training: 2	RegnaTales, Magis
Adaptative cognitive training: 2	EVO Project, ACE
Health behaviour modification strategies: 2	Triumf, PuzzleWalk
Attention bias modification training: 1	Personal Zen
Attention training: 1	Raw Hand

###  Overview of RCT and pilot RCT studies

**Table:** 

Ref	Year	Psych. Approach	Serious Game(s) name	Psychological target	N	Experimental conditions	Main findings
[76]	2016	NM	ACE, EVO Project	Depression	1096	SG vs Problem solving therapy vs Daily Health tips app	Feasability The findings indicate that mobile randomized control trials can quickly and inexpensively recruit a large number of participants. However, engaging the participants in the study remains a difficult task
[68]	2016	NM	Relax and Race The Loom	Stress	50	SG vs VG	Effectiveness Serious Game-based intervention leads to a significant reduction in stress, indicating its effectiveness in teaching users how to manage their stress
[75]	2016	NM	NM	Emotion regulation	61	Computer vs SG vs RGB-D camera	Effectiveness Serious games can be valuable for teaching emotional regulation strategies to non-clinical adolescents. Furthermore, the results emphasize the significance of the interface device in the efficacy of these games, with user interface embodiment influencing users' emotional experience
[70]	2018	CBT	Raw Hand	OCD	30	SG in OCD participants vs SG in health participants	Effect After 3 weeks of gameplay, OCD patients showed increased brain connectivity between the dACC and prefrontal cortex, suggesting that serious games may improve OCD symptoms. Responders had even stronger connectivity to the right superior frontal gyrus compared to nonresponders
[72]	2018	NM	I Love Breast	Emotional disturbance due to medical procedure or illness	76	SG vs Convenional education	Effect The study highlights the feasibility and potential benefits of using smartphone mobile games for breast cancer patients undergoing chemotherapy. Patient education through mobile games resulted in improved drug compliance, reduced side effects, and better quality of life compared to conventional methods. Mobile games can offer easy, enjoyable, and effective ways to educate patients, potentially leading to enhanced treatment outcomes
[69]	2019	NM	Happify Breather	Prevention/promotion well-being	140	No phone vs Phone present but not use vs SG	Effectiveness Participants who engaged in the HRVB game showed lower levels of salivary alpha amylase during recovery compared to other conditions, indicating the effectiveness of the Breather app in improving physiological stress recovery. However, there were no significant differences in salivary cortisol levels or self-reported stress during recovery among the conditions. These findings provide preliminary evidence for the potential of the Breather app in managing stress
[73]	2020	CBT	Drug Defense	Substance /use	140	SG vs Video documentary	Effectiveness The study revealed a significant difference in alcohol knowledge between the game and video settings, with the game having a greater effect size, indicating that repetition aids in transferring information into long-term memory. The game's narrative and interactive elements likely contributed to increased engagement among players, leading to higher scores compared to the control condition (treatment as usual)
[74]	2022	NM	POD Adventures	Prevention/pomotion well-being	11	SG vs Digital flyer	Feasability Acceptability The current results differ significantly from the findings of a previous school-based study of POD Adventures delivered offline before the COVID-19 pandemic. The previous study had higher self-referral and intervention completion rates. Participants found the app helpful in problem-solving and easy to use in the offline setting, with counselors' brief guidance being perceived as effective whether in individual or group sessions
[71]	2022	NM	PuzzleWalk	Neurodevelopmental Disorders	24	SG vs Comercial app	Effectiveness The study's findings revealed that the PuzzleWalk group spent significantly more time using the app compared to the Google Fit group. Additionally, anxiety showed negative associations with increases in light physical activity (PA) and decreases in sedentary time after the intervention
[127]	2022	ACT	Magis	Prevention/promotion well-being	106	SG vs Wait list	Effectiveness The results indicate that higher psychological flexibility correlated with fewer emotional and behavioral problems, improved health-related quality of life, mood, and school satisfaction, as well as reduced loneliness. Although no significant effect was observed in the entire sample, the subgroup of children with initially high psychological inflexibility experienced benefits from participating in the intervention

## Supplementary Information

Below is the link to the electronic supplementary material.

Supplementary file1 (XLSX 65 KB)

## Data Availability

The authors confirm that the data supporting the findings of this study are available within the article and its supplementary materials.

## Footnotes

1 This word was added to the search query after analyzing the keyword frequency of the previously included articles to refine search in the databases.
